# Photoclick chemistry led to the identification of HELLS as a helicase for DNA G-quadruplexes

**DOI:** 10.1093/nar/gkag034

**Published:** 2026-01-22

**Authors:** Zi Gao, Yiewoon Chong, Xiaomei He, Jun Yuan, Yinsheng Wang

**Affiliations:** Department of Chemistry, University of California Riverside, Riverside, CA 92521-0403, United States; Biophysics Graduate Program, University of California Riverside, Riverside, CA 92521-0403, United States; Department of Chemistry, University of California Riverside, Riverside, CA 92521-0403, United States; Department of Chemistry, University of California Riverside, Riverside, CA 92521-0403, United States; Department of Chemistry, University of California Riverside, Riverside, CA 92521-0403, United States; Biophysics Graduate Program, University of California Riverside, Riverside, CA 92521-0403, United States

## Abstract

Guanine quadruplexes (G4s) are unique secondary structures of nucleic acids with functions in many biological processes. Understanding the functions of DNA G4s requires knowledge about their recognition by cellular proteins. Here, we developed a method involving photoclick chemistry and LC-MS/MS-based quantitative proteomics for uncovering G4-binding proteins (G4BPs). By incorporating a photoactivatable *ortho*-nitrobenzylamine moiety into G4 DNA probes and employing UVA irradiation along with stringent washing, we identified 99 proteins enriched with G4 structures derived from the human telomere. By employing fluorescence anisotropy, fluorescence resonance energy transfer, immunofluorescence microscopy, and ChIP-seq analyses, we demonstrated the abilities of one of these proteins, HELLS, in binding and resolving G4 structures *in vitro* and in chromatin. We also found that HELLS-G4 interaction modulates gene expression. Together, we developed a photoclick chemistry-based approach for identifying G4BPs. The approach allowed for the identification of a number of new G4BPs, and we also documented novel functions of one of these proteins, i.e. HELLS, in recognizing and unwinding G4 structures *in vitro* and in cells.

## Graphical Abstract

**Graphical Abstract:**
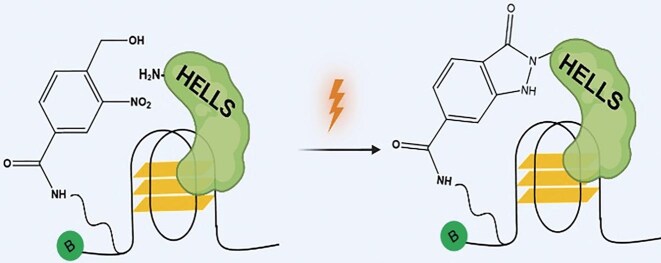

## Introduction

Guanine quadruplexes (G4s) are non-canonical nucleic acid structures comprised of at least three layers of guanine quartets, where guanine bases are interconnected through Hoogsteen base pairing and stabilized by monovalent metal ions [1]. These unique secondary structures have attracted considerable attention owing to their functions in various biological processes, including telomere maintenance [2] as well as DNA replication [3], transcription [4], and repair [5]. Loss of G4 homeostasis has been implicated in a spectrum of human diseases ranging from cancer to neurodegenerative disorders, underscoring the importance of precisely defining the molecular mechanisms governing G4s in cells [6]. This regulatory functions of G4 structures relies critically on proteins that specifically recognize, stabilize, and/or resolve these structures. Aberrant interactions of G4s with certain helicases, e.g. BLM [7], WRN [8], and FANCJ [9], are implicated in genetic disorders. In addition, altered expression of several G4-binding proteins (G4BPs), including FUS [10] and hnRNPA1 [11], has been observed in neurodegenerative diseases. Together, these observations underscore the importance of identifying G4BPs and understanding how G4–protein interactions modulate gene expression and contribute to human diseases.

We previously employed affinity pull-down with biotin-conjugated DNA probes capable or incapable of folding into G4 structures, together with stable isotope labeling by amino acids in cell culture (SILAC)-based quantitative proteomics, to identify G4BPs [12], and we validated the abilities of several identified G4BPs in binding directly with G4 DNA [12–14, 15]. The approach, nevertheless, has some limitations in identifying those proteins exhibiting weak and/or transient interactions with G4 structures. Additionally, this method may also result in the discovery of proteins interacting indirectly with G4 DNA through protein–protein interactions.

Photoaffinity labeling has been employed for identifying and characterizing proteins that can bind to nucleic acids, small molecules, and other proteins [16–21]. In this vein, photo-crosslinking probes relying on G4-binding ligands were employed for identifying G4BPs. For instance, Su *et al.* [22] developed a G4 ligand-mediated cross-linking and pull-down (G4-LIMCAP) method by utilizing a probe consisting of a derivative of a G4-binding ligand, *N,N'*-bis(2-quinolinyl)pyridine-2,6-dicarboxamide, a.k.a. pyridostatin (PDS). This probe encompasses a diazirine group capable of photo-crosslinking and an alkyne group for subsequent Cu(I)-catalyzed azide-alkyne cycloaddition. Similarly, Zang *et al.* [23] reported a co-binding-mediated protein profiling method, which utilizes PDS conjugated with a linker, a diazirine, and an alkyne functionality. Both probes photo-crosslink with G4BPs in cells, where a biotin group can be attached to the probe using click chemistry through the alkyne handle for subsequent avidin-based enrichment. However, because PDS binds to both DNA and RNA G4 structures [24], neither method discriminates DNA from RNA G4BPs. In addition, PDS may compete with some G4BPs in binding toward DNA G4, as documented for YY1 [14], VEZF1 [15], CBS [25], and LARK [26]; thus, the method may not allow for the identification of these G4BPs. Moreover, upon photoactivation, the diazirine moiety undergoes conversion to a reactive carbene intermediate, which can form covalent bonds with nearby biomolecules or be quenched by the surrounding aqueous environment [27]. This may give rise to non-specific interactions with proteins and confer reduced crosslinking efficiency.

Inspired by recently published studies of Guo *et al.* [28, 29] on primary amines and *o*-nitrobenzyl alcohols cyclization (PANAC) photoclick reaction to capture proteins, here, we developed a photoclick chemistry-based method for proteome-wide discovery of G4BPs. With this method, we were able to identify a number of novel G4BPs, including HELLS. We also found that recombinant HELLS protein can bind to and unfold DNA G4 structures *in vitro*, and that the protein modulates G4 structures in chromatin and regulates gene expression in live cells.

## Materials and methods

### Cell culture

HeLa and U2OS cells were maintained in Dulbecco’s modified Eagle’s medium (Thermo Fisher) supplemented with 10% fetal bovine serum (Thermo Fisher), and 1% penicillin–streptomycin solution (GE Healthcare). The cells were incubated at 37°C in a 5% CO_2_ environment.

### Syntheses and characterizations of *o*-NBA-conjugated DNA probes

The G4-forming oligodeoxynucleotides (ODNs) with sequences derived from the human telomere, with a biotin label on the 5′ termini and an aliphatic amine-derivatized thymidine, and the corresponding mutated sequences incapable of folding into G4 structures were purchased from Integrated DNA Technologies (IDT). The conjugation of *ortho*-nitrobenzylamine (*o*-NBA) with ODNs was carried out using a hexafluorophosphate azabenzotriazole tetramethyl uronium (HAUT)-based coupling, following previously described procedures [29]. In brief, the ODN sample was mixed with an equal volume of 500 mM sodium borate (pH 9.4). A mixture containing equal concentrations of HAUT, 4-hydroxymethyl-3-nitrobenzoic acid and *N,N*-diisopropylethylamine (DIPEA) (200 mM each in dimethylacetamide) was prepared and added to the ODN sample. The reaction mixture was incubated on a thermomixer at room temperature overnight. To the resulting solution were subsequently added a solvent mixture of 5 M aqueous solution of NaCl and absolute ethanol (1:2.5, v/v), and the mixture was incubated at −20°C for 1 h to precipitate the ODN. Lastly, the sample was buffer exchanged into water using ultrafiltration (3 kDa MWCO centrifugal filter). The products were subjected to LC-MS and MS/MS analysis on an LTQ linear ion-trap mass spectrometer (Thermo Fisher).

The *o*-NBA-modified G4 and M4 probes were annealed in a buffer containing 10 mM Tris-HCl (pH 7.5), 10 mM KCl, and 0.1 mM EDTA by heating to 95°C for 5 min, followed by cooling down to room temperature slowly over 6 h.

### Photoaffinity labeling

The nuclear proteome was prepared from HeLa cells cultured in [^13^C_6_,^15^N_2_]-Lys- and [^13^C_6_]-Arg-containing medium (heavy) or the corresponding unlabeled Lys- and Arg-containing medium (light) [13] using the NE-PER nuclear and cytoplasmic extraction reagents (Thermo Fisher Scientific), following the manufacturer’s instructions. Protein concentrations were determined using the Bradford Quick Start Protein Assay kit (Bio-Rad).

For the pull-down experiments, 0.5 μM of the aforementioned *o*-NBA-conjugated DNA probes were incubated individually 500 μg of nuclear lysate in a binding buffer (20 mM HEPES, pH 7.5, 100 mM KCl, and 1 mM MgCl_2_) at 4°C with rotation for 30 min. The mixture was then transferred to a 24-well plate and exposed to UVA light on ice for 10 min. A Spectroline XX-15 lamp equipped with two 25-cm long, 15-W light tubes emitting at 365 nm (Spectronics Corporation, Westbury, NY) was employed for the irradiation, where the distance between the lamp and sample solution was 5 cm. Subsequently, the samples were collected and incubated with pre-washed streptavidin beads in the binding buffer at 4°C with rotation for 2 h. After the incubation, unbound proteins were removed by washing three times with a washing buffer containing 20 mM HEPES (pH 7.5), 500 mM NaCl, and 0.1% sodium dodecyl-sulfate (SDS). The beads were subsequently combined, and proteins were eluted from the beads by boiling in a 2 × SDS–polyacrylamide gel electrophoresis (PAGE) loading buffer (Bio-Rad) for 5 min. The resulting eluent was then subjected to in-gel digestion, following a previously published protocol [12, 30], and LC-MS/MS analysis.

### LC-MS/MS analysis

On-line LC-MS/MS analysis of the peptide samples was performed on an Orbitrap Fusion Lumos tribrid mass spectrometer equipped with a Flex nanoelectrospray ion source, which was coupled with a high-field asymmetric-waveform ion mobility spectrometry (FAIMS) and an EASY-nLC 1000 system (Thermo Fisher Scientific). The mass spectrometer was operated in the positive-ion mode with a spray voltage of 2.0 kV. The compensation voltages for the FAIMS were set at −40, −60, and −80 V, and the carrier gas flow rate was set at 4.2 l/min. Cycle time of each compensation voltage was set to 1 s. The HPLC separation was performed using a trapping column followed by an analytical column, both packed in-house with ReproSil-Pur C18-AQ resin with particle sizes of 5 and 3 µm, respectively (Dr Maisch HPLC GmbH, Germany). The peptides were separated using a 160-min linear gradient of 8%–34% acetonitrile in 0.1% formic acid at a flow rate of 300 nl/min. Full-scan MS (*m/z* 300–1200) were acquired at a resolution of 60 000, followed by MS/MS acquisition in the linear ion trap, where the scan rate was set as rapid. Fragmentation was conducted with higher-energy collisional dissociation at a fixed collisional energy of 30%. The mass spectrometry proteomics data were deposited to the ProteomeXchange Consortium via the PRIDE [31] partner repository with the dataset identifier PXD051342.

### Proteomics data analysis

Protein identification and quantification were conducted by searching the raw LC-MS and MS/MS data using MaxQuant (Version 2.1.2.0) [32] against the UniProt human proteome database (Proteome ID: UP000005640_9606). For quantification at MS level, the multiplicity was set to 2, and Lys8 and Arg6 were selected as heavy amino acids. Cysteine carboamidomethylation was set as a fixed modification, along with N-terminal protein acetylation and methionine oxidation being set as variable modifications. The maximum number of missed cleavages for trypsin was set to two per peptide, and peptide length range was 7–25 amino acids. The tolerances in mass accuracy for MS and MS/MS were 20 ppm and 0.5 Da, respectively. The match between runs option was enabled with the alignment window being 3 min.

### Plasmid construction and protein purification

The coding sequence of human HELLS was amplified from the pBac-HELLS plasmid, which was generously provided by Dr Kathrin Muegge at the National Cancer Institute [33]. Subsequently, the coding sequence was inserted into the pRK7 vector, incorporating 3 × Flag epitope tag at the carboxyl terminus. HEK293T cells were transfected with the plasmid using TransIT-2020 (Mirus Bio, Madison, WI) and harvested 36 h later for protein extraction. CelLytic M Cell Lysis Reagent (C2978, Sigma) supplemented with 1 × protease inhibitor cocktail (P8340, Sigma) was used for protein extraction. Protein purification was conducted according to the manufacturer’s instructions. Briefly, the cell lysate was incubated with Anti-Flag M2 magnetic beads (M8823, Sigma) at 4°C for 1 h, followed by washing with tris-buffered saline (TBS) (50 mM Tris–HCl, pH 7.4, 150 mM NaCl), and the Flag-tagged HELLS protein was eluted from the beads with 3 × Flag peptide (NC0792928, Thermo Fisher Scientific).

### Circular dichroism spectroscopy

DNA probes of 5′-T G4, 5′-T M4, Loop-T G4, and Loop-T M4 (10 μM each) were annealed in a binding buffer containing 20 mM HEPES (pH 7.5), 100 mM KCl, and 1 mM MgCl_2_ by heating to 95°C in a water bath, followed by cooling slowly to room temperature for over 3 h. The circular dichroism (CD) spectra were recorded as an average of three scans in the wavelength range of 200–320 nm on a Jasco-815 spectropolarimeter. The spectra were baseline-corrected using the corresponding buffer.

### Fluorescence anisotropy

Fluorescence anisotropy measurements were conducted using a microplate reader (Synergy H1, Agilent). 5′-TAMRA-labeled DNA (2 nM final concentration) was incubated with different concentrations of recombinant HELLS in a buffer containing 20 mM HEPES (pH 7.5), 50 mM KCl, 2.5 mM MgCl_2_, and 0.5 mM dithiothreitol (DTT). Anisotropy was recorded using the red filter set with the excitation and emission wavelength being 530 and 590 nm, respectively.

### Fluorescence resonance energy transfer assay

Fluorescence resonance energy transfer (FRET)-based unwinding assay was performed according to previously published procedures [34]. FAM-MycG4-BHQ1 and Cy3-hTelG4-BHQ2 DNA probes (20 μM) were annealed in a 40 mM Tris-acetate buffer (pH 7.5) containing 100 mM LiCl or KCl by heating to 94°C, followed by gradual cooling to room temperature over 3 h. The unwinding assay was conducted using 20 nM G4 probe dissolved in a buffer containing 50 mM Tris-acetate (pH 7.5), 50 mM KCl, 2.5 mM MgCl_2_, 0.5 mM DTT, and the indicated concentrations of HELLS protein. The fluorescence measurements were carried out at room temperature using a Horiba PTI QM-400 fluorescence spectrophotometer. The excitation wavelengths for FAM-MycG4-BHQ1 and Cy3-hTelG4-BHQ2 DNA probes were 495 and 405 nm, respectively, and the emission spectra were collected in the wavelength ranges of 510–550 and 415–600 nm, respectively.

### Western blot

For detection of HELLS knockdown efficiency, U2OS cells were lysed with CelLytic M cell lysis reagent (Sigma) supplemented with 1% protease inhibitor cocktail. Protein concentration was measured by Quick Start Bradford Protein Assay (Bio-Rad) and denatured at 95°C for 5 min in a SDS–PAGE loading buffer (Bio-Rad). The lysates were separated using SDS–PAGE and transferred onto a nitrocellulose membrane. The membrane was blocked with 5% non-fat milk in phosphate buffered saline (PBS)-T for 1 h and then incubated with the corresponding primary antibodies, including HELLS polyclonal antibody (Proteintech, 11955-1-AP, 1:500 dilution) and anti-tubulin (Santa Cruz, SC-32293, 1:5000). The secondary antibodies were donkey anti-rabbit secondary antibody (Sigma, A0545, 1:5000) or anti-mouse secondary antibody (Santa Cruz, m-IgGκ BP-HRP, 1:5000). The western blot signal was detected using ECL western blotting detection reagent (Amersham, Little Chalfont, UK) and imaged in a LI-COR Odyssey system.

### Immunofluorescence

Immunofluorescence microscopy experiments were conducted according to previously established protocols [35, 36]. In brief, control and HELLS knockdown cells were fixed in a mixture of methanol and acetic acid (3:1, v/v) at room temperature for 10 min. Subsequently, the cells were permeabilized using 0.1% triton-X100 in 1 × PBS for 15 min on ice, followed by treatment with RNase A (Thermo Fisher Scientific) at 37°C for 30 min. After blocking with 2% bovine serum albumin (BSA) at room temperature for 1 h, the immunofluorescence microscopy experiments were carried out using standard methods. BG4 antibody (MABE917, Sigma–Aldrich), anti-FLAG (14793S, Cell Signaling Technology), and anti-rabbit Alexa 594-conjugated secondary antibody (A11037, Invitrogen) were used. Nuclei were stained using DAPI (D9542, Sigma–Aldrich). Finally, the coverslips were mounted with ProLong Diamond Antifade Mountant (Invitrogen). Images were captured using an LSM880 confocal laser scanning microscope (Carl Zeiss) equipped with a 100 × objective, and subsequent image analysis was performed using ZEN software. The number of foci per nucleus was quantified using the Find Maxima feature in ImageJ.

### Reverse transcription-quantitative PCR

U2OS cells expressing shControl, shHELLS-1, and shHELLS-2 were cultured in six-well plates until reaching ∼50% confluency, and then treated with 20 μM PDS for 15 h. Total RNA was extracted using TRIzol reagent and quantified. Reverse transcription was performed using SuperScript IV reverse transcriptase to generate complementary DNA library. Reverse transcription-quantitative polymerase chain reaction (RT-qPCR) was carried out with Luna® Universal qPCR Master Mix (NEB) on a CFX96 real-time detection system (Bio-Rad). Primer sequences for the target genes, including *MXD1, ING1*, and *HSPA1B*, are provided in Supplementary Table S1.

### G4-ChIP-seq and qPCR

G4-ChIP was conducted by following previously described procedures with slight modifications, using the custom-purified BG4 antibody [36]. Briefly, the chromatin samples were sonicated and diluted in a blocking buffer containing 25 mM HEPES (pH 7.5), 10.5 mM NaCl, 110 mM KCl, 1 mM MgCl_2_, and 1% BSA, and treated with RNase A. Subsequently, the chromatin samples were incubated with 500 ng of the BG4 antibody under rotation at 1400 rpm for 1.5 h at 16°C. Pre-blocked Anti-Flag M2 magnetic beads (Sigma, M8823) were added to the mixture, which was then incubated under the same conditions for 1 h. After washing the beads with ice-cold washing buffer (10 mM Tris, pH 7.4, 100 mM KCl, 0.1% Tween 20) for six cycles, the DNA was eluted with TE buffer containing proteinase K while rotating at 1400 rpm for 6 h at 65°C. The eluted DNA was purified using the DNA Clean and Concentrator-5 kit (Zymo). qPCR was performed using Luna Universal qPCR Master Mix (NEB) on a CFX96 touch real-time PCR detection system (Bio-Rad). The primer sequences are listed in Supplementary Table S1.

Chromatin immunoprecipitation-sequencing (ChIP-seq) library was prepared using the NEBNext® Ultra™ II DNA Library Prep Kit (NEB) following the manufacturer’s instructions. The purified DNA libraries were subsequently quantified using an Agilent 2100 Bioanalyzer and multiplexed for sequencing at Genomics Research and Technology Hub (Nova-seq 6000, GRT Hub, UC Irvine) with 100-bp paired-end sequencing. Raw reads of ChIP-seq data were aligned to human hg38 reference genome using Bowtie2 (v2.4.5) [37]. PCR duplication was removed by Picard MarkDuplicates. Unique reads were filtered using Samtools [38]. Heatmap and metagene profile were generated by deeptools (v3.5.1) [39] plotHeatmap and plotProfile, respectively. Genome coverage bigwig files for Integrative Genomics Viewer visualization were generated by deeptools (v3.5.1) [39] bamCoverage using RPGC for the normalization.

### Bioinformatics analysis

Overlapping analysis of HELLS ChIP-seq and BG4 ChIP-seq data was performed using bedtools intersect command with default parameters [40]. Monte Carlo simulation was conducted using gkmSVM to generate GC content- and length-matched null sequence in mm9 genome background [41]. A total of 100 null peaksets were generated, and overlapping analysis with BG4 ChIP-seq was performed. Annotation of overlapped regions was performed using HOMER annotatePeaks.pl with default setting [42].

## Results

### Preparation and characterizations of *o*-NBA-modified G4 photoclick probes

**Figure 1.**
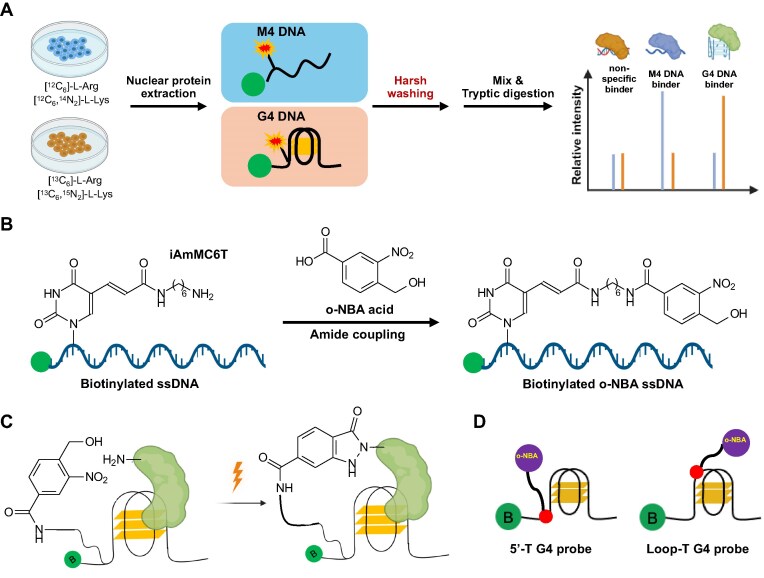
Photoclick chemistry together with SILAC-based quantitative proteomics for the identification of G4BPs. (**A**) A schematic diagram showing the experimental workflow. (**B**) The chemical structures of biotinylated G4-ODNs with a modified thymidine derivative containing an internal amino modifier C6 dT (iAmMC6T), and the structure of the *o*-NBA photo-activating group conjugated through amide coupling. (**C**) The ɛ-amino group of lysine residue in G4BPs in close proximity can be captured by *o*-NBA group after photoactivation. (**D**) *o*-NBA group was deliberately placed on thymidines at two different positions, i.e. on the 5′ side and in the loop region of the G4 structure, namely, 5′-T and Loop-T G4 probes, respectively.

The experimental procedures for SILAC-based quantitative proteomic screening were similar to our previously reported method [12, 13], albeit with important modifications (Fig. 1A). In particular, an *o*-NBA group is conjugated with the C5 of a modified thymine derivative in the biotin-labeled G4 DNA probe derived from the human telomere through amine coupling (Fig. 1B). A six-carbon straight-chain alkane linker is inserted between the *o*-NBA-moiety and thymidine, which provides flexibility for PANAC reaction with primary amines in the proximal proteome [28] (Fig. 1C). We also synthesized the corresponding mutated sequence (M4) incapable of folding into G4 structures and used it as a control. Recognizing that G4BPs can interact with different regions of G4 structures, we designed two pairs of probes, namely, the 5′-T G4 probe with the *o*-NBA group being placed on the thymidine near the 5′-terminus of the G4 structure and the Loop-T G4 probe with the *o*-NBA group being installed on the thymidine in the loop region of the G4 structure (Fig. 1D). The successful syntheses of these DNA probes were verified by LC-MS/MS analysis (Supplementary Fig. S1). We also confirmed that the incorporation of *o*-NBA-modified thymidine does not perturb G4 folding, where the CD spectra for the G4 probes are very similar to those published previously (Supplementary Fig. S2) [13, 43].

### Proteome-wide identification of G4-binding proteins using PANAC photoclick chemistry

In the affinity pull-down experiments, the G4 and M4 probes were incubated with the light- and heavy-SILAC-labeled nuclear protein lysates, respectively, followed by irradiation with 365-nm light to induce crosslinking. The irradiation mixture was incubated with streptavidin beads, and the beads were washed thoroughly with a buffer containing 500 mM NaCl and 0.1% SDS to remove non-covalently bound proteins. The beads containing the heavy- and light-labeled proteins were subsequently combined. The proteins were eluted from the beads, digested with trypsin, and the ensuing peptides subjected to LC-MS/MS analysis.

**Figure 2.**
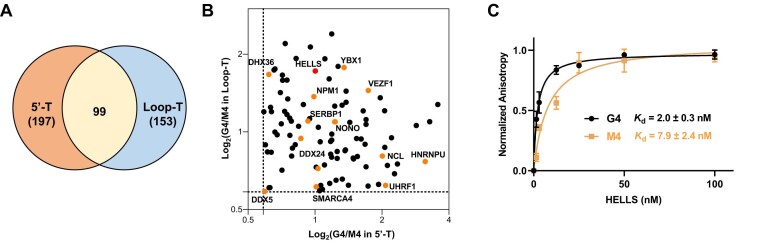
Identification of HELLS as a novel G4BP. (**A**) A Venn diagram showing the overlap of putative G4BPs identified from the two sets of pull-down experiments with G4/M4 > 1.5. (**B**) A scatter plot of the common putative G4BPs identified from pull-down experiments using the 5′-T and Loop-T G4 probes. Previously reported G4BPs are highlighted in orange. (**C**) Fluorescence anisotropy for monitoring the binding of HELLS with 5′-TAMRA-labeled G4 and M4 DNA probes.

Using a cutoff SILAC ratio (G4/M4) of ≥1.5, we identified 296 and 252 candidate G4BPs with the 5′-T and Loop-T probes, respectively (Figs 2A and S3A and B; Supplementary Table S2). Among the identified G4BPs, 99 were commonly identified with both G4 probes, and they included previously reported G4BPs, e.g. DHX36 [44], nucleolin [45], and VEZF1 [15] (Supplementary Table S2 and Fig. 2B). Gene ontology analysis revealed that these candidate G4BPs are highly enriched in molecular functions of helicase activity, chromatin binding, catalytic activity acting on a nucleic acid, etc. (Supplementary Fig. S3D), consistent with biological processes associated with G4 structures and functions of known G4BPs. These data support that the photoclick chemistry approach captures physiologically relevant G4-interacting protein factors. Quantitative enrichments obtained from the two probes exhibit good correlation, demonstrating the robustness of the method (Supplementary Fig. S3C). In addition, the identification of probe-specific interactors suggests that distinct spatial positioning of the *o*-NBA moiety can differentially capture subsets of G4BPs, likely reflecting genuine differences in protein engagement with different G4 structural elements. Within this set of confidently identified G4BPs, HELLS (helicase, lymphoid-specific) displays strong and reproducible enrichment across both probes, exhibiting SILAC ratios of 3.3 (5′-T) and 2.0 (Loop-T) (Supplementary Figs S3A–B and S4). HELLS possesses intrinsic DNA- and nucleosome-stimulated enzymatic activity and plays essential roles in nucleosome remodeling through unwinding DNA in nucleosome [46, 47]. Given its important functions and its robust enrichment in both G4 proteomic datasets, we selected HELLS for subsequent studies.

### HELLS binds G4 DNA and unwinds G4 structures *in vitro*

To gain insights into the interaction between HELLS and DNA G4 structures, we purified recombinant Flag-tagged HELLS protein from HEK293T cells (Supplementary Fig. S5A) and employed fluorescence anisotropy to measure the binding affinities between HELLS and G4 DNA. The results revealed a strong and preferential binding of HELLS toward G4 DNA over the mutated single-stranded DNA (M4), with the dissociation constants (*K*_d_) being 2.0 ± 0.3 and 7.9 ± 2.4 nM, respectively (Fig. 2C). The *in vitro* binding result validated our proteomics findings and revealed HELLS as a G4BP.

**Figure 3.**
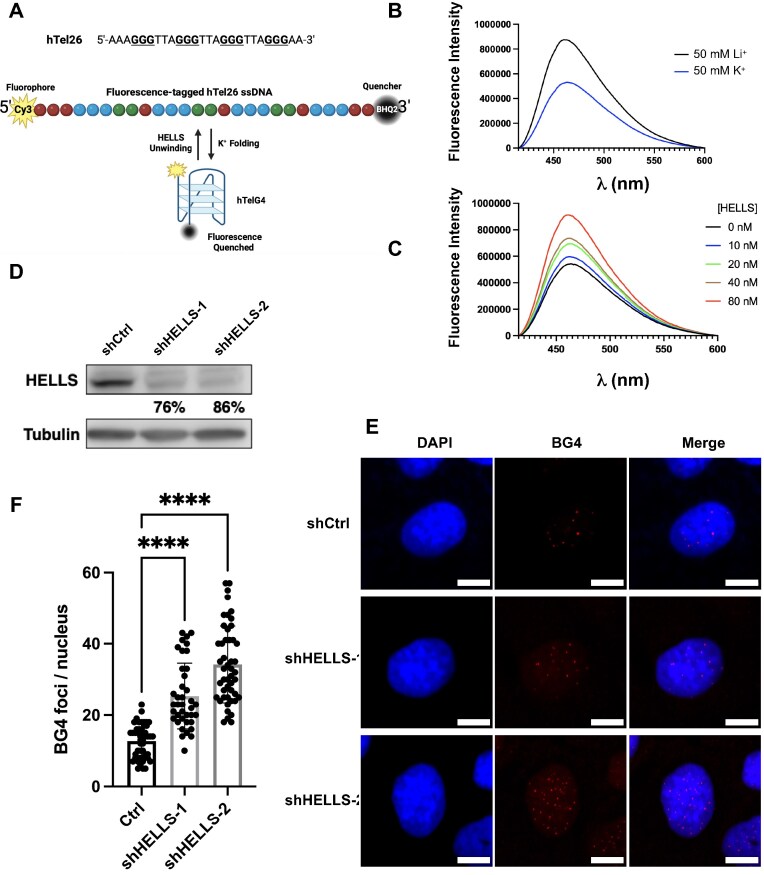
HELLS unwinds G4 structures *in vitro* and in cells. (**A**–**C**) FRET assay showing that HELLS unwinds DNA G4 structures derived from human telomere *in vitro*. (**D**) Western blot for monitoring the knockdown efficiencies of HELLS in U2OS cells with two different sequences of shRNAs. (**E**) Immunofluorescence microscopy images of G4s in control and HELLS knockdown cells (scale bar: 10 µm). (**F**) Quantification data of the numbers of G4 foci per nucleus in cells treated with shCtrl and shHELLS. The *P* values were calculated using one-way analysis of variance (ANOVA) (****P < .0001)

We next assessed the ability of HELLS in unwinding DNA G4 structures *in vitro* by employing a previously reported FRET assay [34]. In particular, a Cy3 fluorophore and a quencher (BHQ-2) are conjugated with the 5′- and 3′-termini of the telomere G4 DNA probe, respectively (Fig. 3A). In this vein, when the DNA adopts a folded G4 conformation (such as that stabilized in K⁺ buffer), fluorescence is quenched, whereas G4 unfolding increases the distance between fluorophore and quencher, resulting in enhanced fluorescence emission. As expected, maximal fluorescence signal was observed in Li⁺ buffer, while a quenched baseline fluorescence was observed in K⁺ buffer, which is consistent with the unfolded and folded G4 structures in Li^+^ and K^+^ buffers, respectively (Fig. 3B). Upon addition of increasing concentrations of recombinant HELLS, we observed a concentration-dependent increase in fluorescence emission, demonstrating that HELLS resolves G4 structures *in vitro* (Fig. 3C). Similar results were obtained from another G4 sequence derived from *MYC* promoter (Supplementary Fig. S5). The G4 sequences derived from human telomere and *MYC* promoter are known to adopt hybrid and parallel G4 topologies, respectively [48, 49], underscoring the abilities of HELLS in unwinding various types of G4 structures.

### Depletion of HELLS led to increased accumulation of G4 structures in cells

We next examined the roles of HELLS in modulating G4 structures in cells. To this end, we established U2OS cells with stable knockdown of HELLS using short-hairpin RNA (shRNA), where the successful knockdown of HELLS was confirmed by western blot analysis (Fig. 3D). Next, we monitored the levels of G4 structures in cells using immunofluorescence microscopy with the BG4 antibody [35]. Our results revealed a > 2-fold increase in global G4 levels upon HELLS knockdown, where a higher knockdown efficiency confers a greater number of G4 foci in cells (Fig. 3E and F). This observation underscores that the G4-resolving activity of HELLS observed *in vitro* has functional relevance in a cellular context.

### HELLS co-localizes with G4 structure sites and unwinds G4 structures in chromatin

HELLS was previously shown to modulate chromatin architecture and transcription [50, 51]. Notably, HELLS assumes a pivotal role in facilitating the binding of transcription factors to chromatin, especially in promoter regions [52]. To gain a comprehensive understanding about the interaction of HELLS with G4 structures in chromatin at the genome-wide scale, we performed bioinformatic analysis of publicly available ChIP-seq data of HELLS acquired for mouse embryonic fibroblasts [52] and G4 ChIP-seq data for mouse embryonic stem cells [53].

**Figure 4.**
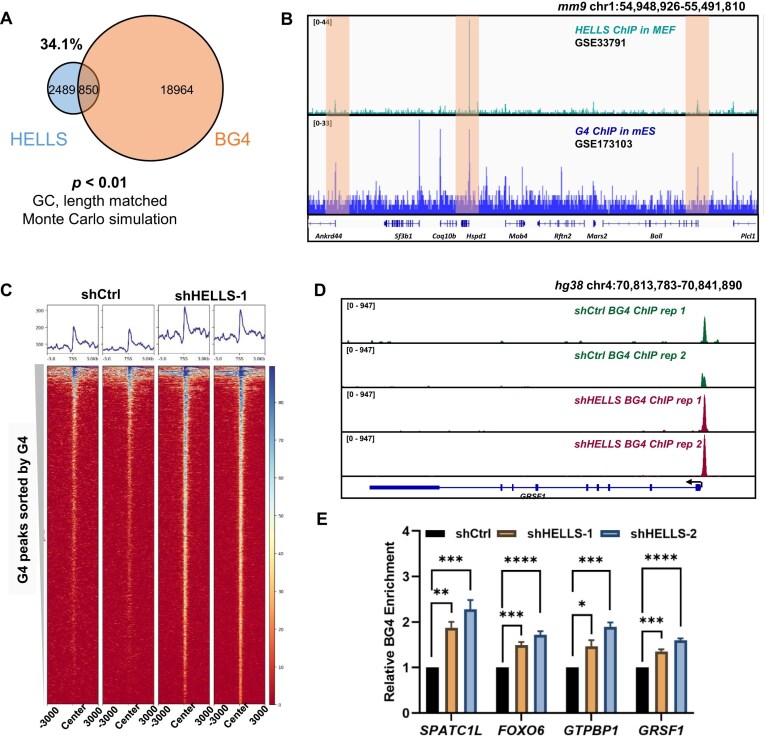
HELLS unwinds G4 structures in cells. (**A**) A Venn diagram showing overlapping peaks obtained from publicly available HELLS-ChIP-seq and BG4-ChIP-seq datasets in mouse cells. (**B**) Representative IGV (Integrative Genomics Viewer) plots showing the co-localization of HELLS binding sites with G4 structure loci in chromatin. (**C**) Heat maps showing enrichment of BG4-ChIP-seq signal on G4 loci in shCtrl and shHELLS U2OS cells. Heat maps are ranked according to G4 enrichment in shCtrl cells in descending order. (**D**) Representative IGV plots showing that HELLS knockdown led to an increase in BG4-ChIP signal in promoter regions. (**E**) BG4-ChIP-qPCR results showing that knockdown of HELLS resulted in augmented enrichment of G4 structures in the promoter regions of *GRSF1, FOXO6, GTPBP1*, and *SPATC1L* genes. *, .01 < *P* < .05; **, .001 < *P* < .01; ***, .0001 < *P* < .001; ****, *P* < .0001. The *P* values were calculated using one-way ANOVA.

Such analysis unveiled a substantial co-occupancy of HELLS at G4 structure loci. In particular, ~34.1% of HELLS ChIP-seq peaks overlapped with BG4 ChIP-seq peaks (Fig. 4A and B). We also performed Monte Carlo simulations to estimate the significance of such overlapping. In this vein, we generated 100 null peaksets with the same number of peaks, similar GC content and similar length distribution, and then performed the same overlapping analysis with BG4 ChIP-seq data. The result demonstrated that none of them out-compete HELLS ChIP-seq dataset, suggesting that HELLS selectively targets G4-associated loci rather than simply GC-rich DNA. In this context, it is worth noting that the two ChIP-seq datasets were generated in two different cell lines and the distribution of G4 structures in chromatin is cell type-dependent [54]. Hence, this likely underestimates the extent of overlap when compared to the situations where the HELLS and BG4 ChIP-seq experiments are conducted using the same cell line, though we cannot exclude the possibility that some of overlaps revealed herein may not exist in other cell types. Moreover, the majority of overlapped peaks are mapped to promoter regions (Supplementary Fig. S6B), consistent with the known roles of HELLS in promoter-associated chromatin remodeling [55].

To test directly whether HELLS modulates G4 structures in human cells, we performed BG4 ChIP-seq in U2OS cells following shRNA-mediated depletion of HELLS. Our results revealed an elevated accumulation of G4 structures in U2OS cells upon shRNA-mediated knockdown of HELLS, which is in keeping with what we observed in the immunofluorescence experiment. Importantly, we observed increased number of BG4 ChIP-seq peaks in promoter regions upon HELLS knockdown (Fig. 4C–E and Supplementary Fig. S6C), In addition, results from bioinformatic analysis of BG4 ChIP-seq data showed that BG4 ChIP-seq peaks detected in shControl and shHELLS cells showed high percentages of G4-forming sequences (Supplementary Fig. S6A), where the G4-forming sequences were determined using G4hunter [56]. We also validated the ChIP-seq results by conducting ChIP-qPCR experiments to monitor G4s in promoter regions of several genes (*FOXO6, GRSF1, GTPBP1*, and *SPATC1L*) in U2OS cells (Fig. 4E). It turned out that HELLS depletion resulted in elevated accumulation of G4 structures at all examined loci, where a higher knockdown efficiency conferred by shHELLS-2 elicited more pronounced accumulations of G4 at these loci than shHELLS-1. Collectively, these findings revealed a role of HELLS in dynamic modulation of G4 structures in cells by unwinding these structures.

### HELLS regulates G4-dependent transcription

**Figure 5.**
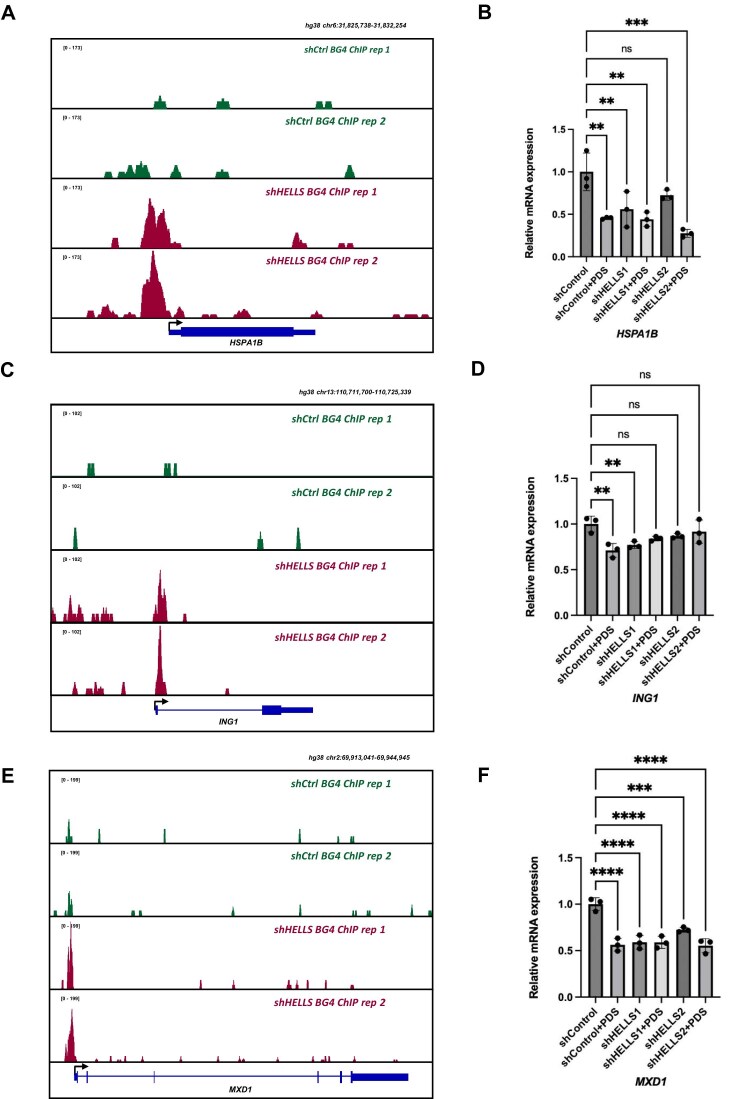
HELLS-mediated unwinding of DNA G4 structures modulates gene expression in cells. (**A, C, E**) Representative IGV plots showing that knockdown of HELLS resulted in increased accumulation of G4 structures in the promoter regions of *HSPA1B, ING1*, and *MXD* genes; (**B, D, F**) Genetic depletion of HELLS and treatment of cells with PDS led to diminished expression of these genes. $\beta $-actin was used as the reference. Error bars represent mean ± SEM. ns, *P* > .05; *, *P *< .05; **, *P *< .01; ****, *P *< .0001 (*n* = 3). The *P* values were calculated using one-way ANOVA.

We next asked whether HELLS-dependent G4 resolution influences transcription. To this end, we measured the expression of several genes whose promoters exhibit elevated enrichment of G4 structures upon HELLS knockdown. RT-qPCR analysis revealed that loss of HELLS led to reduced expression of these genes (Fig. 5). We also treated cells with PDS, reasoning that if HELLS-dependent transcription is G4-sensitive, transcriptional alterations emanating from PDS-medicated stabilization of G4s should phenocopy those arising from HELLS depletion. Indeed, PDS treatment similarly reduced expression of these target genes (*HSPA1B, ING1, MXD1*) in control cells, whereas PDS exposure conferred minimal additional effects in HELLS-depleted cells (Fig. 5). Together, these results support a model in which HELLS-mediated G4 unwinding enhances transcriptional output from those genes enriched with G4 structures in their promoters.

## Discussion

In this work, we developed a photoclick chemistry-based platform that enables covalent capture and proteomic discovery of proteins interacting with DNA G4 structures. By integrating site-specific incorporation of *o*-NBA into G4 DNA probes with SILAC-based quantitative proteomics, this strategy overcomes several limitations of previously established G4BP discovery methods, including limited ability to trap weak or transient interactions and the use of exogenous G4 ligand that may compete with G4BPs in binding with G4 DNA. In addition, photoclick activation enables controlled UVA-triggered cyclization with proximal primary amines in proteins, providing covalent stabilization of G4–protein complexes, enabling stringent washing without loss of *bona fide* G4BPs while minimizing pulling down indirectly bound proteins.

Application of this platform led to an expanded G4BP landscape, providing candidate G4BPs for future studies. We also showed that one of these proteins, i.e. HELLS, directly binds and unwinds G4 structures *in vitro* and in cells, suppresses G4 accumulation in chromatin, and modulates transcriptional output from those genes enriched with G4 structures at their promoters.

Aberrant expression and function of HELLS are associated with various diseases, including cancer and immunodeficiency disorders [33, 57]. For instance, mutations in *HELLS* gene result in immunodeficiency, centromeric instability, and facial anomalies [58]. Therefore, HELLS has emerged as an important target for therapeutic interventions of cancer. HELLS is also known to facilitate transcription factor binding to chromatin, particularly at promoters. It interacts with regulatory proteins such as p53, DNMT1, and HP1 to modulate DNA methylation, histone post-translational modifications, and chromatin structure [57, 59, 60]. Furthermore, HELLS actively participates in DNA replication, where it prevents replication fork stalling and promotes efficient replication [50], and it is also involved in homologous recombination-mediated DNA damage repair [60]. Given the broad relevance of HELLS dysregulation in human diseases, our findings offer new entry points to investigate G4-dependent pathogenesis and suggest targeting G4 as a potential therapeutic strategy.

A limitation of our approach is that covalent capture is performed in lysates using exogenous probes, which may not fully preserve endogenous higher-order G4–protein complexes. As a result, certain context-dependent interactions may be underrepresented. Nonetheless, this strategy is highly powerful for proteome-scale discovery of high-confidence candidate G4BPs and provides a foundation for targeted mechanistic studies in native cellular settings. Finally, beyond G4 biology, with our success in applying this photoclick chemistry-based probe design in G4, the chemical principle can be readily extended to other structured nucleic acids, such as i-motifs, triplex DNA, and other non-canonical structures of DNA. Thus, the platform presented here lays a foundation for systematic and structure-specific mapping of nucleic acid–protein interactions at the proteome-wide scale, enabling future discovery of structure-dependent regulatory networks embedded in the genome.

## Supplementary Material

gkag034_Supplemental_File

## Data Availability

The G4-ChIP-seq data generated in this study have been deposited into the NCBI GEO database with accession number GSE263592. The LC-MS/MS data were deposited to the ProteomeXchange (PXD051342).
