# Supplementary material for: Photoclick chemistry led to the identification of HELLS as a helicase for DNA G-quadruplexes
Source: Nucleic Acids Res. 2026 Jan 22;54(3):gkag034. doi: 10.1093/nar/gkag034 (PMC12825294; doi:10.1093/nar/gkag034)

Supplementary Materials for

"Photoclick Chemistry Led to the Identification of HELLS as a Helicase for DNA G-quadruplexes"

Zi Gao[1,‡], Yiewoon Chong[2,‡], Xiaomei He[1,‡], Jun Yuan[1,‡] and Yinsheng Wang[1,2,‡,*]

[1]Department of Chemistry and [2]Biophyics Graduate Program, University of California Riverside, Riverside, CA 92521-0403.

[‡]These authors contributed equally.

*To whom correspondence should be addressed: yinsheng.wang@ucr.edu

Table of Contents

Table S1. A list of G4 DNA sequences and RT-qPCR/ChIP-qPCR primers used in this study.

Table S2. A list of potential G4-binding proteins identified in this study.

Figure S1. ESI-MS of DNA probes.

Figure S2. Circular dichroism spectra of *o*-NBA-labeled thymidine-containing 5'-T and Loop-T probes used for the quantitative proteomic experiment.

Figure S3. Putative G4-binding proteins in two sets of pulldown experiments.

Figure S4. HELLS binds preferentially to G4 structures over the mutant M4 sequences.

Figure S5. SDS-PAGE gel image of the recombinant Flag-tagged HELLS protein, and FRET assay for assessing the ability of HELLS in unwinding G4 structure derived from the promoter of *MYC* gene.

Figure S6. BG4 ChIP-seq revealed elevated accumulation of G4 structures in the promoter regions and 5′-UTR of genes. (A) Percentage of BG4 ChIP-seq peaks containing G4-forming sequence calculated using G4Hunter mapper identified from cells treated with shCtrl and shHELLS. (B) Gene annotation of overlapping peaks between HELLS-ChIP-seq and BG4-ChIP-seq datasets. (C) IGV plots showing that HELLS knockdown led to augmented BG4-ChIP signal in the promoter regions of *FOXO6* and *GTPBP1* genes.

Table S1. A list of G4 DNA sequences and RT-qPCR/ChIP-qPCR primers used in this study.

**DNA sequences in pulldown experiments**		
5′-T G4	5′-Biotin-T6 /iAmMC6T/AAAGGGTTAGGGTTAGGGTTAGGGAA-3’	
5′-T M4	5′-Biotin-T6 /iAmMC6T/AAAGGGTTAGTGTTAGTGTTAGGGAA-3’	
Loop-T G4	5′-Biotin-T6 TAAAGGGTTAGGGT/iAmMC6T/AGGGTTAGGGAA-3’	
Loop-T M4	5′-Biotin-T6 TAAAGGGTTAGTGT/iAmMC6T/AGTGTTAGGGAA-3’	
**Fluorophore-labeled DNA**		
G4 DNA	5'- TAMRA-TGAGGGTGGGGAGGGTGGGGAAGG-3'	
M4 DNA	5'- TAMRA-TGAGGGTGAGGAGTGTGGGGAAGG-3'	
hTelG4	5'-Cy3-AAAGGGTTAGGGTTAGGGTTAGGGAA-BHQ2-3'	
MycG4	5'-FAM-TGAGGGTGGGGAGGGTGGGGAAGG-BHQ1-3'	
**RT-qPCR primers**		
Genes	Forward	Reverse
HSPA1B	5'-CATCAGCGGACTGTACCAG-3'	5'-TCCACCTCAAAGACAAACATACT-3'
ING1	5'-CCTGGTGAACTATGTGGAGG-3'	5'-CTCGTAGCACTCGTCTAGC-3'
MXD1	5'-AGAGATGCCTTAAAACGGAGG-3'	5'-TTGATTCGGGTCCAAGTGG-3'
**ChIP-qPCR primers**		
Genes	Forward	Reverse
GRSF1	5'-GGGCTGCCCATGGTTTG-3'	5'-TCAGTTTCCACCGCCTGA-3'
FOXO6	5'-GGCACAAAGAAGAGCAGAGT-3'	5'-GACAGGATCCCTCCCAGAA-3'
GTPBP1	5'-TATCTCCATGAGCAGGTCCA-3'	5'-CAACGGAAGCAGAGAAGTGT-3'
SPATC1L	5'-CACCCACCACCACCAAC-3'	5'-GCACCTGAGTGCCCTTG-3'

Table S2. A list of potential G4-binding proteins identified in this study. 'NaN' represents data not available.

Gene Name	5'-T ratio (G4/M4)		Loop-T ratio (G4/M4)	
	**Avg.**	**S.D.**	**Avg.**	**S.D.**
MYO1C	5.27	3.73	2.28	NaN
DDX54	4.60	3.68	1.66	NaN
MORC2	4.39	NaN	1.94	NaN
MKI67	3.80	3.10	1.77	0.40
XPC	3.79	0.02	2.15	0.54
YBX1	3.47	2.71	2.41	1.32
FBL	3.42	2.31	2.55	0.48
GNL2	3.37	3.20	1.58	0.88
ZNF638	3.34	NaN	1.57	NaN
HELLS	3.30	1.20	2.00	0.01
DHX36	3.18	1.58	1.54	0.89
RPL4	3.16	2.24	1.64	0.67
RPL17	3.04	NaN	2.23	0.31
MYO1B	3.00	1.39	1.68	NaN
SFPQ	3.00	2.27	1.64	0.29
VEZF1	2.73	NaN	3.33	NaN
ZC3H14	2.71	NaN	2.60	NaN
GNL3	2.68	1.72	1.62	0.35
NPM1	2.58	1.82	1.98	0.41
PSIP1	2.56	1.97	3.91	1.57
KIF2A	2.53	NaN	1.69	NaN
THAP11	2.47	NaN	2.60	NaN
TOP2A	2.42	1.56	1.85	0.42
LRPPRC	2.42	0.71	11.84	4.55
DDX49	2.41	NaN	2.50	0.21
SF3B1	2.39	1.23	2.34	0.53
GTPBP4	2.37	1.44	1.55	NaN
FBLL1	2.31	1.00	4.16	NaN
KIF22	2.31	1.91	3.13	NaN
TMPO	2.30	1.46	1.66	0.47
CALD1	2.29	1.45	1.55	0.25
MYBBP1A	2.29	1.33	1.50	0.26
RPS3A	2.25	0.83	1.68	0.68

RRP1B	2.19	1.32	1.70	0.88
POLRMT	2.18	0.36	1.93	0.30
DDX18	2.17	1.15	3.24	0.51
SERBP1	2.15	1.93	1.90	0.60
ATAD3A	2.14	1.19	1.72	0.29
GTF3C5	2.14	0.03	9.70	1.48
NONO	2.14	1.10	2.34	0.80
SF3B2	2.13	0.67	2.35	1.94
SUGP2	2.13	0.03	2.70	0.65
PRPF8	2.11	1.02	1.63	0.72
NCAPH2	2.10	0.44	3.24	1.28
XRCC5	2.08	0.17	7.22	2.35
TMEM201	2.07	NaN	1.79	NaN
TOP2B	2.06	1.18	2.05	0.23
RFC1	2.00	1.18	1.58	0.09
RECQL	1.97	0.78	2.28	0.05
SPTBN1	1.97	0.79	1.60	0.34
CSDE1	1.96	0.87	1.71	1.14
MTA2	1.93	1.06	4.62	1.37
DDX24	1.92	0.98	1.82	NaN
TRIM28	1.92	0.79	2.90	0.29
POLR1E	1.92	0.82	3.06	NaN
EXOSC10	1.90	0.63	2.28	0.61
LBR	1.89	0.57	2.26	1.83
IGF2BP3	1.89	0.94	2.08	0.32
ZC3HAV1	1.86	1.48	3.20	1.90
RRP7A	1.86	1.25	1.76	NaN
NOP56	1.85	0.81	1.51	0.34
PHF6	1.83	0.81	4.17	0.93
SUPT5H	1.82	0.56	1.73	0.45
PRRC2A	1.80	1.11	2.59	1.40
SCAF11	1.80	0.35	2.57	1.02
SMC4	1.79	0.63	2.39	NaN
CEBPZ	1.79	0.78	1.53	0.06
UTP20	1.78	0.82	1.55	0.25
SART1	1.76	0.89	2.26	NaN
NCL	1.75	0.88	4.02	0.68
SEC61A1;SEC61A2	1.75	0.99	1.72	0.69
BCLAF1	1.74	0.69	2.93	0.29

TEX10	1.74	0.66	2.28	0.55
ABCF1	1.73	0.81	2.80	1.19
RIF1	1.73	0.72	2.39	0.58
MDC1	1.72	0.90	2.81	0.75
HNRNPU	1.70	0.85	8.74	5.01
GTF3C3	1.68	0.05	5.08	1.20
THOC2	1.67	0.56	2.15	0.67
GRWD1	1.67	0.65	4.33	0.89
LARP1	1.66	0.78	3.24	1.20
ILF3	1.66	0.41	2.02	0.49
LAS1L	1.65	0.54	2.04	0.26
KIF4A	1.64	0.84	1.64	0.22
ZNF629	1.61	0.54	4.85	NaN
NCAPD3	1.58	0.52	2.51	0.72
BAZ1B	1.57	0.62	2.37	0.54
SMC2	1.55	0.47	2.24	0.33
GTF3C1	1.54	0.17	3.81	0.79
POLR2A	1.54	NaN	2.66	NaN
CPSF1	1.54	0.12	2.09	0.85
UHRF1	1.54	0.36	4.22	0.64
POLR1B	1.53	NaN	3.32	NaN
SMARCA4	1.53	0.43	2.02	0.50
RCC2	1.52	0.67	1.54	0.38
PELP1	1.52	0.74	1.54	0.58
PRKDC	1.51	0.46	2.11	0.82
PDCD11	1.51	0.54	2.07	1.50
DDX5	1.50	0.75	1.51	0.18

Figure S1. ESI-MS showing the $[M - 10H]^{10-}$ ions of the 5′-T *o*-NBA G4 (A) or M4 (B); Loop-T *o*-NBA G4 (C) or M4 (D).

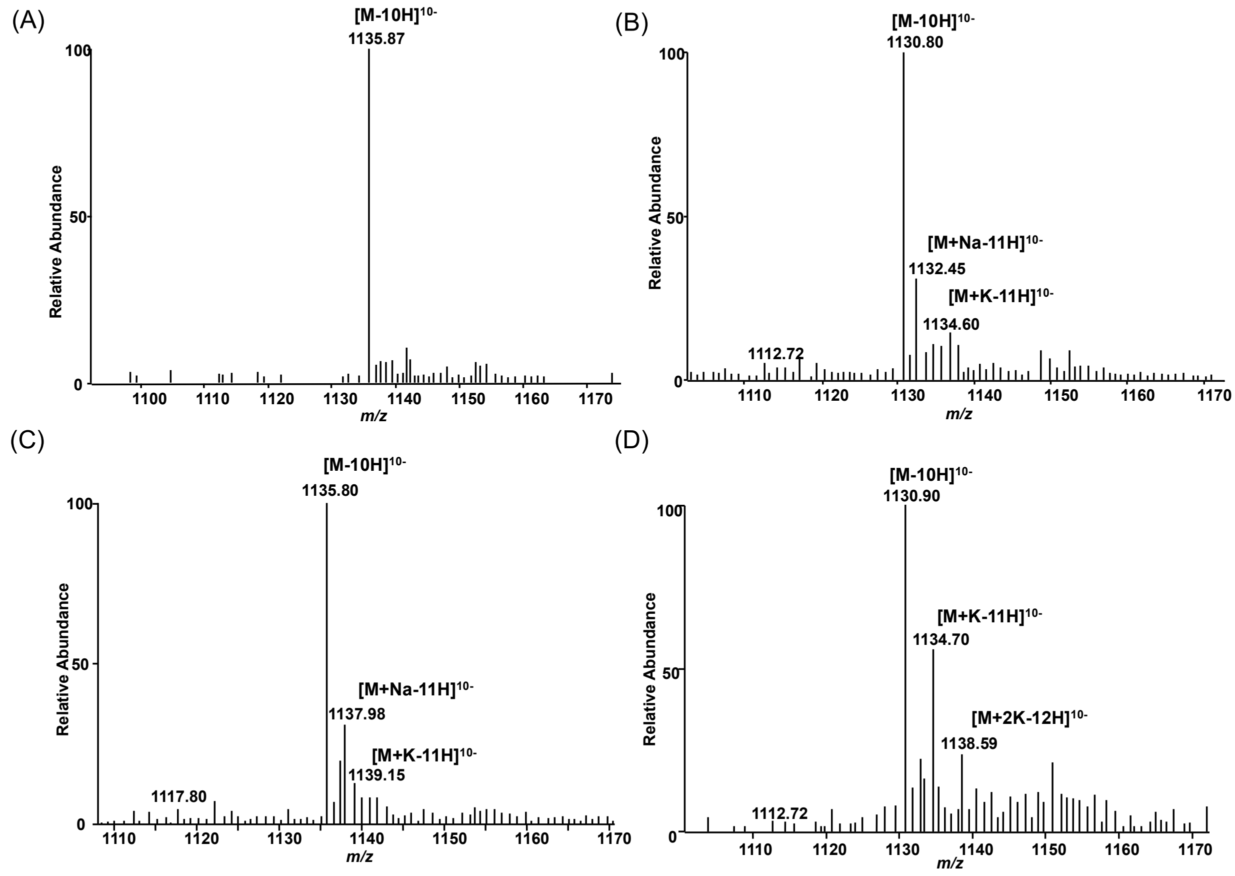

Figure S2. Circular dichroism spectra of *o*-NBA-labeled thymidine-containing 5'-T (A) and Loop-T (B) probes used for the quantitative proteomic experiment.

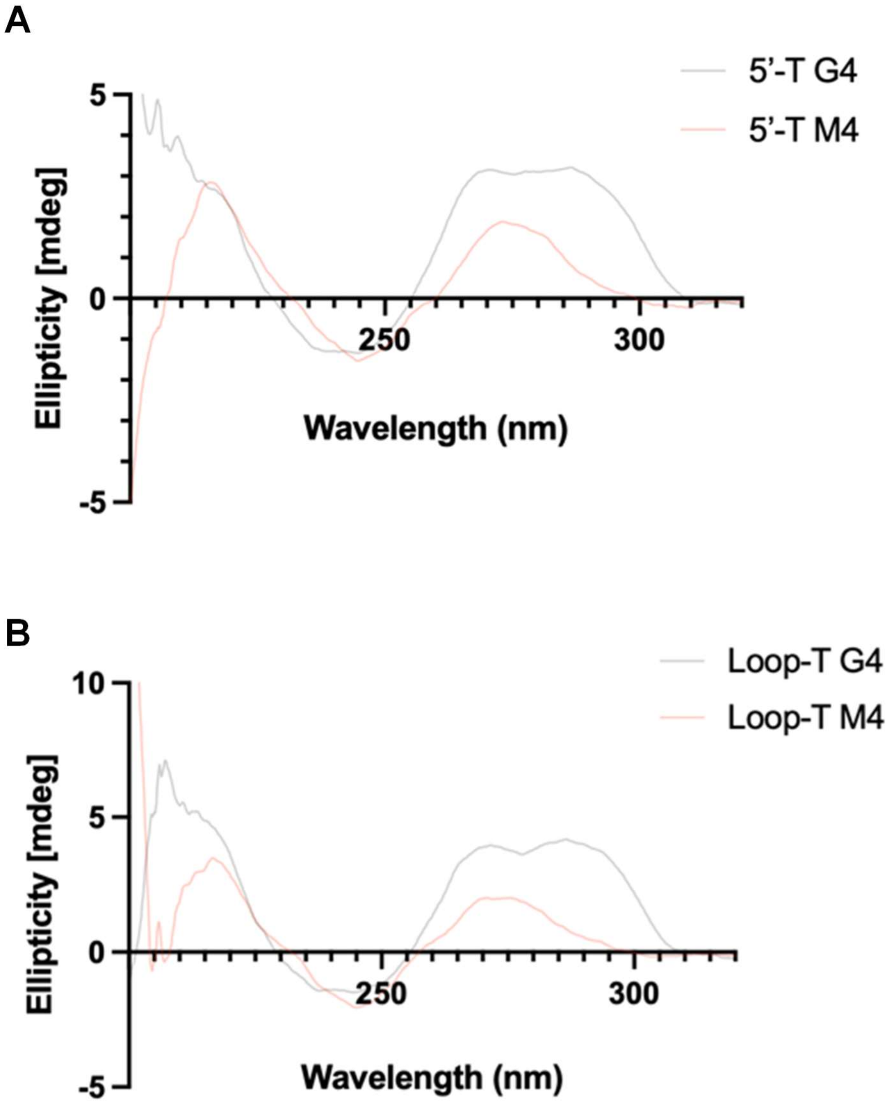

Figure S3. Putative G4-binding proteins identified from two sets of pull-down experiments. Volcano plots illustrating the identified G4-binding proteins in pull-down experiments using probes with the *o*-NBA group placed at the thymidine on G4 loop region (Loop-T) (A) or near the 5′-terminus (5′-T) (B). (C) A scatter plot displaying the protein ratio of G4/M4 of commonly detected protein in both sets of pull-down experiments. (D) Gene ontology (GO) analysis showing enriched molecular functions, biological processes and cellular components associated with the identified G4BPs.

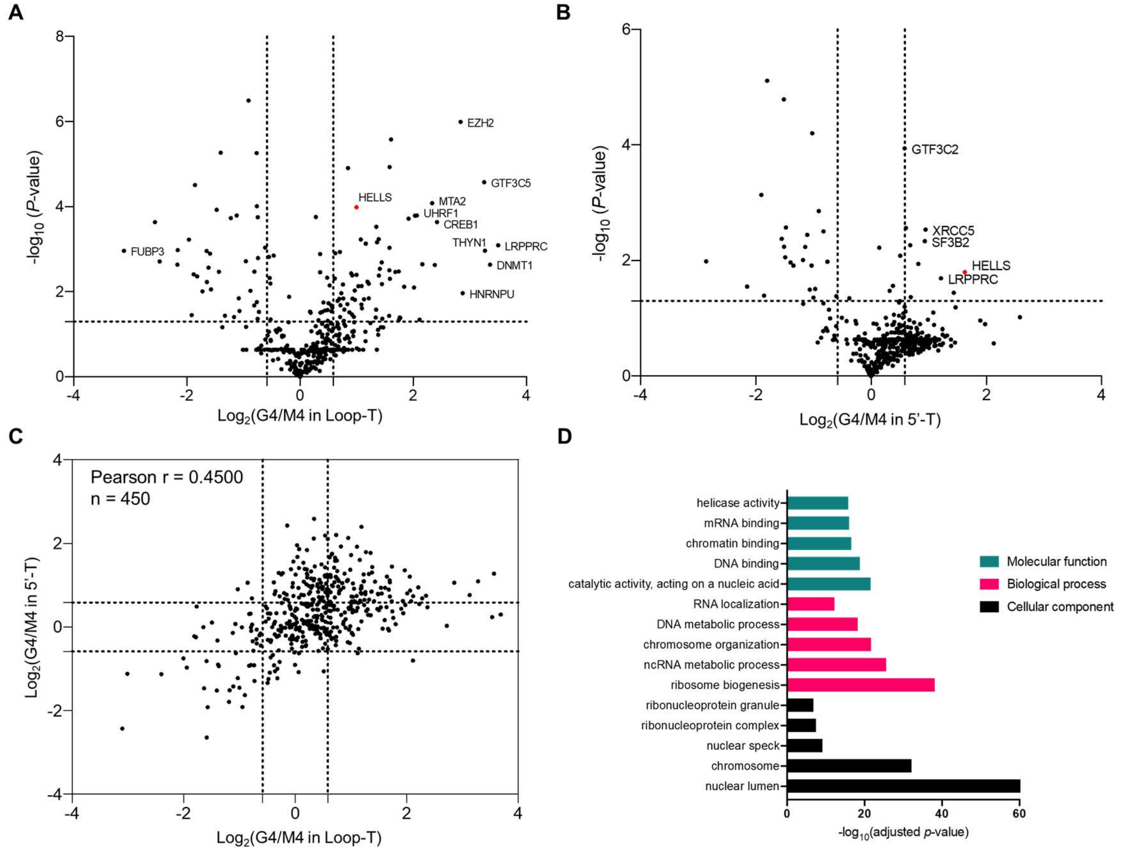

Figure S4. HELLS binds preferentially to G4 structures over the mutant M4 sequences. (A-D) ESI-MS showing the $[M + 2H]^{2+}$ ions of light and heavy lysine-containing peptide LVTANTIDQK with the monoisotopic *m/z* values being ~ 551.81 and 555.81, respectively, obtained from forward (A, C) and reverse (B, D) SILAC-based photo-crosslinking experiments using Loop-T (A B) and 5′-T (C, D) probes. (E, F) MS/MS for the $[M+2H]^{2+}$ ions of the light (E) and heavy (F) lysine-containing peptide, LVTANTIDQK.

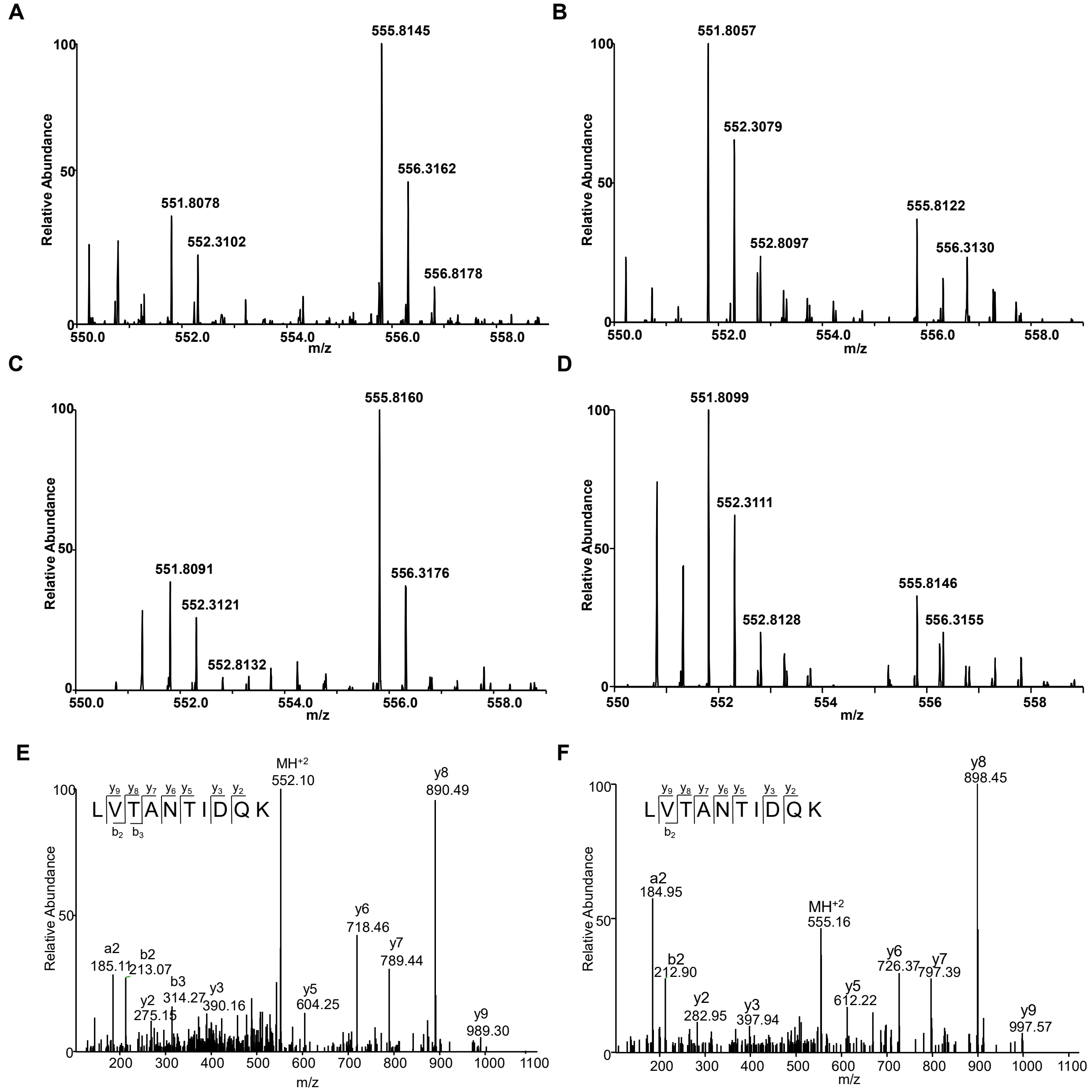

Figure S5. Purification of recombinant HELLS protein and FRET for assessing the ability of HELLS in unwinding G4 structure derived from the promoter region of *MYC* gene *in vitro*. (A) SDS-PAGE showing the purity of the recombinant Flag-tagged HELLS protein. (B) A schematic diagram showing the FRET assay for examining the ability of HELLS in unwinding G4 structure derived from the *MYC* promoter. (C-D) Fluorescence emission spectra showing the impact of K^+ and Li^+ ions (C) and different concentrations of HELLS protein in modulating the folding of the G4 structure derived from the *MYC* promoter sequence.

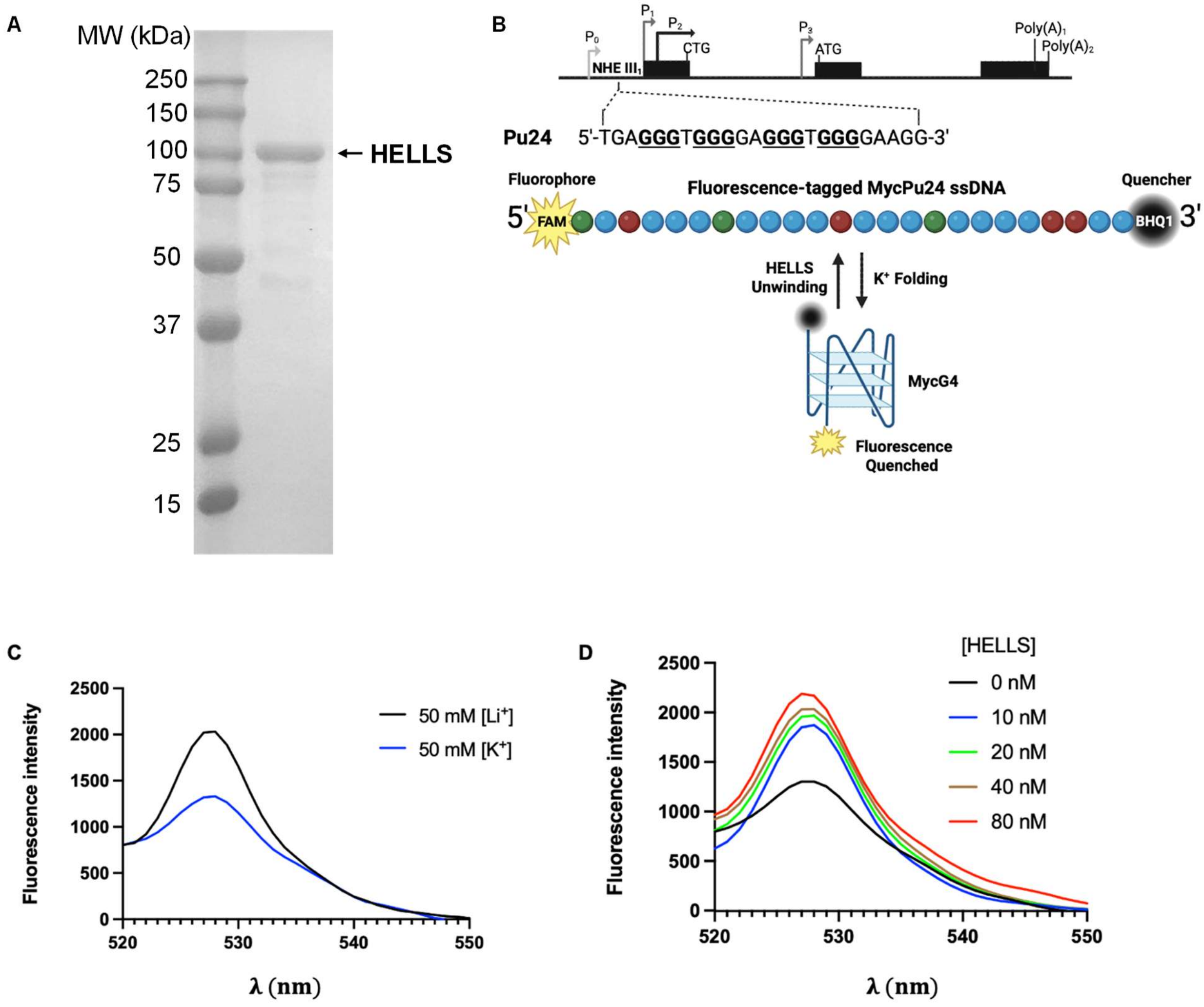

Figure S6. BG4 ChIP-seq revealed elevated accumulation of G4 structures in the promoter regions and 5′-UTR of genes. (A) Percentage of peaks containing G4-forming sequence calculated using G4Hunter mapper in shCtrl and shHELLS ChIP-seq. (B) Gene annotation of overlapping peaks between HELLS-ChIP-seq and BG4-ChIP-seq datasets. (C) IGV plots showing that HELLS knockdown led to augmented BG4-ChIP signal in the promoter regions of *FOXO6* and *GTPBP1* genes.

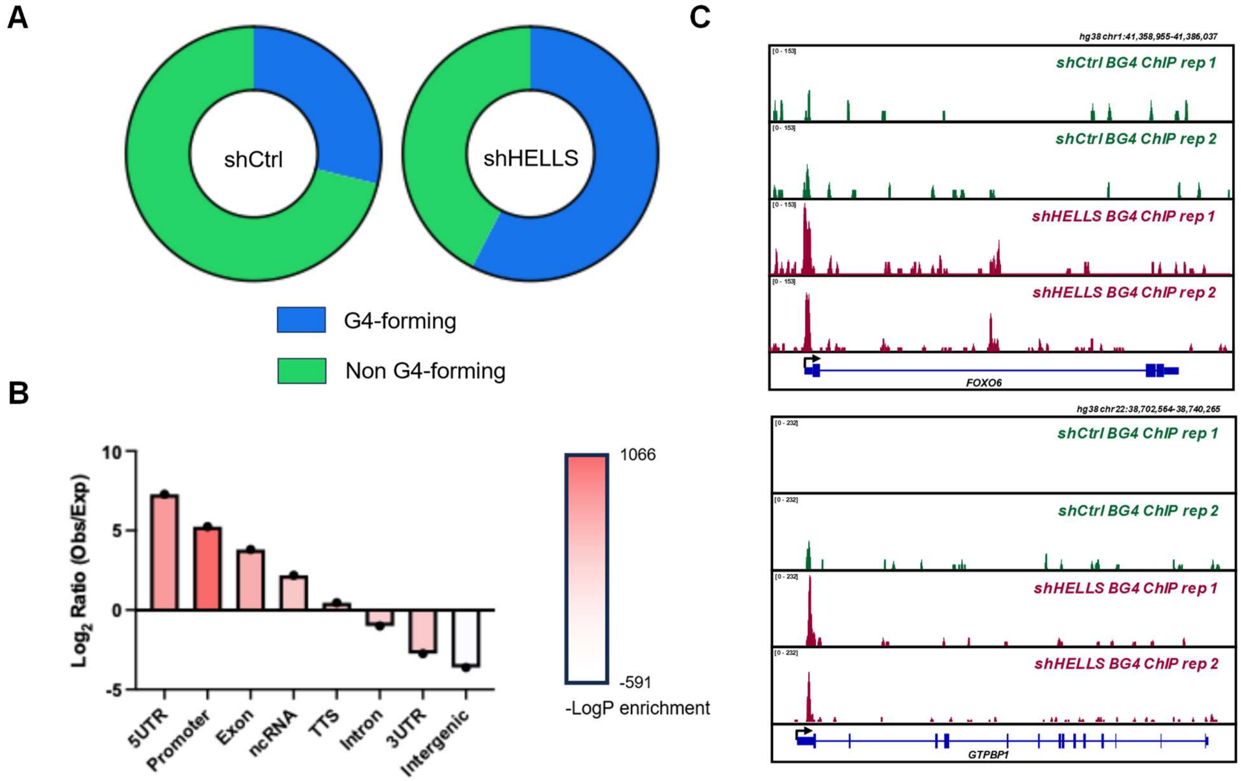

Supplement: gkag034_Supplemental_File [file gkag034_supplemental_file.pdf]
